# The Validity of Sleeping Hours of Healthy Young Children as Reported by Their Parents

**DOI:** 10.2188/jea.12.237

**Published:** 2007-11-30

**Authors:** Michikazu Sekine, Xiaoli Chen, Shimako Hamanishi, Hongbing Wang, Takashi Yamagami, Sadanobu Kagamimori

**Affiliations:** 1 Toyama Medical and Pharmaceutical University.; 2 Hokuriku Health Service Association.

**Keywords:** validity, sleep, questionnaire, children, parents, Actiwatch^®^

## Abstract

The aim of this study was to evaluate the validity of sleeping hours of young children as reported by their parents. The subjects were 21 healthy children aged 3 to 4 years. They were asked to attach a small instrument for calculating sleeping hours objectively, over 3 consecutive nights. Parents reported the sleeping hours of their children during the study periods. The mean values were used in the analysis. Pearson’s correlation coefficients and paired t-tests were used to evaluate the correlations and differences between the reported and objectively measured sleeping hours. The correlation coefficient and difference between the reported and assumed (objective sleeping hours representing the difference between times for falling asleep and waking) sleeping hours were 0.90 (p<0.001) and 0.79 hours (95% confidence interval: 0.59-0.99), respectively. The correlation coefficient and difference between the reported and actual (the assumed, sleeping hours minus the sum of epochs being scored as awake during the assumed sleep) sleeping hours were 0.90 (p<0.001) and 0.92 hours (0.73-1.10), respectively. Although parents tended to overestimate the sleeping hours of their children, the correlation between the reported and objective sleeping hours is high, which indicates that reported sleeping hours could be used in a survey that requires data on relative differences in sleeping hours amongst a given population.

## INTRODUCTION

The importance of sleeping habits in young children has been increasingly recognized^1^^-^^5^^)^. In addition to the well-known association between sleeping habits and psychiatric problems^1^^-^^3^^)^, several epidemiological studies have recently mentioned the relationship between short sleeping hours and the development of somatic problems such as child obesity^4^^,^^5^^)^.

Many previous epidemiological studies in children have widely used self-reported or parent-reported sleeping habits^1^^-^^7^^)^. However, the validity of the sleeping habits of young children as reported by their parents has not been established. The accuracy of sleeping habits, particularly of young children as reported by their parents, may be unreliable for several reasons. Firstly, sleeping habits dramatically change in preschool years^7^^,^^8^^)^. While typical infantile sleep is characterized by frequent intermittent short sleeps, amounting to 12 hours sleep or more per day, the impact of aging on sleeping habits is characterized by a sizable long sleep and shortening of sleeping hours^7^^,^^8^^)^. Secondly, because parents may tend to report periods between times for going to bed and rising rather than times for falling asleep and waking, sleeping hours as reported by parents may be longer than the accurate sleeping hours of their children as evaluated by electroencephalogram (EEG). Thirdly, different children may have a different quality of sleep^9^^,^^10^^)^, which may result in the misclassification of sleeping hours. For example, the sleeping hours of children with sleeping problems may be characterized by fragmented short sleeps and intermittent waking during the night^9^^,^^10^^)^. Then actual sleeping hours may be much shorter than the period between bedtime and rising time. In considering the detailed background biological mechanisms for the above-mentioned psychiatric and somatic problems, changes in hormonal secretion and autonomic nerve activity may play a crucial role in the development of such diseases^11^^,^^12^^)^. However, these pathophysiological changes are highly dependent on sleep quality and quantity as evaluated by EEG^11^^,^^12^^)^. Therefore, the accuracy of the sleeping hours of young children as reported by their parents should be clarified, in order to apply the data of reported sleeping hours to a large epidemiological study.

The purpose of this study is to evaluate the validity of the sleeping hours of young children as reported by their parents, with the use of a small instrument capable of assessing sleeping hours objectively.

## METHODS

We are currently conducting a birth cohort study comprising of approximately 10000 children born in 1989^5^^,^^13^^)^. The present study was conducted in July and August 2001 as a sub-study of the follow-up study.

### Subjects

An introductory letter was sent to the parents of 55 children aged 3 to 4 years, belonging to a public nursery school in Toyama prefecture. Of those parents, 24 agreed to participate and provided written informed consent. Of the participants, 1 child was reluctant to continue attaching the instrument and we terminated the measurement. Data from the 2 other children were not successfully measured and were excluded in the analysis. The remaining 21 children (12 boys and 9 girls) with a mean age of 3.81 (SD: 0.28) years were the subjects of the present study. These subjects did not have any chronic disease nor any obvious sleeping problem and did not take any medication during the measurement days.

### Anthropometric measurements

All the anthropometric measurements were performed by one examiner. The heights and weights of children were measured wearing shorts. The heights of children were measured using a stadiometer, to the nearest 0.1 cm. The weights of children were measured using a balance scale to the nearest 0.1 kg. Body mass index (BMI; the weight in kilograms divided by the squared height in meters) was calculated as an index of obesity. The anthropometric measurements were conducted twice. The means were used in the analysis.

### Assessment of sleeping habits in children

The sleeping habits of children were assessed by the use of a questionnaire and small instrument capable of assessing sleep quality and quantity.

We asked parents to complete a questionnaire containing the following questions on the sleeping habits of children, for 3 consecutive nights during the measurement days: get up time: when does your child get up? ; bedtime: when does your child go to bed? ; sleeping hours: how long does your child take a sleep? The sleep questionnaire values were expressed in hours and minutes. The means of the values from the 3 nights were used in the analysis.

For the objective measurement of sleeping habits, we used a small instrument capable of assessing sleeping quality and quantity by measuring activity, named Actiwatch^®^ (Mini Mitter Company Inc., Bend, OR)^14^^)^. Detailed information on the methodology for calculating the activity indices by this small instrument has been published elsewhere^14^^)^. In summary, the Actiwatch^®^ is a small (27mm x 26mm x 9 mm in size), light weight (17grams), limb worn, activity and sleep-wake pattern monitoring device, which contains an omni-directional sensor capable of detecting acceleration in two planes with a sensitivity of 0.01 gravity (0.098 m/sec^2^). This sensor integrates the degree and speed of motion and produces an electrical current that varies in magnitude. An increased degree of speed and motion produces an increase in voltage. The instrument stores this information as activity counts. The maximum sampling frequency is 32Hz. In the present study, the instrument was attached to a subjects’ ankle on the non-dominant leg. The subjects and parents were asked to keep the instrument attached for 3 consecutive days. After the researcher has entered bedtime and rising times and selected the length of sampling epoch and the sensitivity threshold, the device automatically estimates various sleep parameters according to the algorithm. We selected a sampling epoch of 1 minute and automatic sensitivity, in which the software automatically scores an epoch as sleep if the total activity value is equal to or less than the threshold sensitivity value calculated by (mean score in activity period x K (constant=0.888)) / (epoch length). The parameters calculated by the instrument are the following: sleep start: time of sleep onset; sleep end: the time of sleep termination; assumed sleeping hours: the difference in time between the end and start of sleep; actual sleeping hours: the assumed sleeping hours minus the sum of the epochs being scored as awake during the assumed sleep; sleep latency: the period between bedtime and sleep start; sleep efficiency: actual sleeping hours divided by the time in bed and multiplied by 100. All values, other than sleep efficiency, are expressed in hours and minutes. The unit of sleep efficiency is %.

The instrument has been validated using polysomnography (PSG) as the reference^15^^-^^17^^)^. The percentages of the agreement of sleep/wake detection between PSG and the instrument ranged from 85 to 92 %^15^^,^^16^^)^. There was a significant positive association between PSG and the instrument for sleep latency and sleep efficiency^17^^)^. The instrument has been used in sleep studies^18^^,^^19^^)^.

### Statistical analysis

The sex differences in the reported and objective sleep parameters and anthropometric data were compared by unpaired t-tests. The differences and correlations between the reported and objective sleeping hours were analysed by paired t-tests and Pearson’s correlation coefficients. All statistical analyses were performed with the SPSS (7.5.1J) statistical package. A two-tailed P value of less than 0.05 was considered to be significant.

## RESULTS

**Figure 1.**
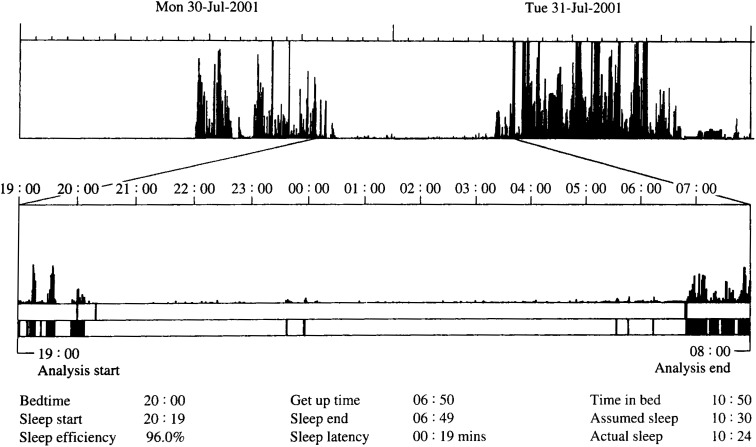
A representative actogram obtained from a subject. A representative actogram obtained from a subject is shown. The spikes in the upper actogram represent the activity counts per epoch. The higher spikes indicate higher activity. The lower actogram is an expanded window from the upper actogram in the nighttime. The solid bars in the lower actogram represent bouts considered as wake, according to the algorithm. Bedtime and get up time were manually entered. The other sleep parameters were automatically calculated.

Figure 1 shows a representative actogram obtained from a subject. The data shows the 2 objective sleeping hours including the assumed and actual sleeping hours were calculated as 10:30 and 10:24, respectively.

Table 1 shows the characteristics of the study subjects by gender. There were no significant differences in ages of children, anthropometric data, and reported and objective sleep parameters between boys and girls. Reported sleeping hours during the measurements, and assumed and actual sleeping hours, as evaluated by the instrument, were 9.42, 8.63, and 8.51 hours, respectively. The mean sleep latency was 26.0 min. The mean sleep efficiency was 90.5%. Data from boys and girls were combined in the later analysis because there were no significant sex differences in the sleeping parameters. Table 2 presents the correlation and difference between the reported and objective sleeping hours. The correlation and difference between the reported sleeping hours during the measurement days and assumed or actual sleeping hours were 0.90 (p<0.001) and 0.79 hours (95%confidence interval: 0.59-0.99) and 0.90 (p<0.001) and 0.92 hours (0.73-1.10), respectively. The correlation and difference between the assumed and actual sleeping hours were 0.99 and 0.12 hours (0.06-0.19), respectively.

**Table 1.** Characteristics of the subjects of the present study.

	boys (n=12)	boys (n=12)	girls (n=9)	p value
	
	Mean (SD)	Mean (SD)	Mean (SD)	
Age and anthropometric data				
age (years old)	3.81 (0.28)	3.79 (0.24)	3.84 (0.33)	0.728
height (cm)	98.8 (3.00)	99.5 (2.98)	97.9 (2.95)	0.236
weight (kg)	15.4 (1.28)	15.5 (1.18)	15.3 (1.47)	0.783
BMI (kg/m^2^)	15.8 (0.78)	15.6 (0.69)	16.0 (0.88)	0.353

Reported sleeping habits during the measurement days				
bedtime (hours:minutes)	21:39 (0:59)	21:43 (0:59)	21:34 (1:01)	0.745
get up time (hours:minutes)	7:04 (0:35)	7:04 (0:38)	7:04 (0:34)	0.993
sleeping hours (hours)	9.42 (0.96)	9.36 (1.16)	9.51 (0.67)	0.734

Objective sleeping habits				
sleep start (hours:minutes)	22:06 (1:02)	22:10 (1:05)	21:59 (1:00)	0.685
sleep end (hours:minutes)	6:44 (0:34)	6:45 (0:34)	6:43 (0:37)	0.900
sleep latency (minutes)	26.0 (17.0)	27.0 (18.0)	25.0 (17.0)	0.733
assumed sleeping (hours)	8.63 (0.95)	8.56 (1.09)	8.73 (0.79)	0.698
actual sleep (hours)	8.51 (0.91)	8.44 (1.04)	8.60 (0.75)	0.700
sleep efficiency (%)	90.5 (4.13)	90.5 (3.75)	90.4 (4.83)	0.965

The values are expressed as mean (SD). The sleep parameters between boys and girls are compared by unpaired t-tests.

**Table 2.** Correlations and differences between the reported and measured sleeping hours.

	correlation coefficients		differences and 95%confidence intervals	
	
	r	p value	difference (95%CI)	p value
reported vs assumed sleeping hours	0.90	<0.001	0.79 (0.59-0.99) ^1^	<0.001
reported vs actual sleeping hours	0.90	<0.001	0.92 (0.73-1.10) ^1^	<0.001
assumed vs actual sleeping hours	0.99	<0.001	0.12 (0.06-0.19) ^2^	<0.001

The correlations and differences between the reported and measured sleeping hours are evaluated by Pearson’s correlation coefficients and paired t-tests. The data are expressed as hours. ^1^The differences between reported and objective sleeping hours are calculated by using objective sleeping hours as a reference. ^2^The differences between assumed and actual sleeping hours are calculated by using actual sleeping hours as a reference. Abbreviations: r: Pearson’s correlation coefficient, 95%CI: 95%confidence interval

## DISCUSSION

The present study indicates that the sleeping hours of young children as reported by their parents are strongly related to the assumed sleeping hours (r=0.90) as estimated by the Actiwatch^®^ instrument. Furthermore, the mean difference between the reported and assumed sleeping hours is small, 0.79 hours (47.4 min). The difference between the reported and assumed sleeping hours may be explained by the sum of the difference between times for going to bed and falling asleep (sleep latency) and the difference between times for waking and rising (early morning waking). Coble et al.^20^^)^ reported that sleep latency and early morning awakening of young children aged 6 to 7 years, as evaluated by EEG, were 18.4 min and 6.3 min on average, respectively. In the present study, sleep latency and early morning awakening were 27 min and 20.4 min, respectively. Therefore, if the difference in experimental settings (laboratory vs field study, etc.) is taken into consideration, our findings are not so inconsistent with those of the previous study.

In the present study, we also evaluated the correlation and difference between the reported and actual sleeping hours. The correlation coefficient was also high (r=0.90), although a slightly larger difference between the reported and actual sleeping hours was observed than the difference between the reported and assumed sleeping hours.

Our results indicate, therefore, that the reported sleeping hours are strongly associated with the objective sleeping hours, although parents tend to uniformly overestimate the sleeping hours of their children. This means that the sleeping hours of healthy young children as reported by their parents could be used to evaluate the relative difference in sleeping hours in healthy young children and could be applied to a large epidemiological survey, which requires data of relative differences in sleeping hours amongst a given population.

In the present study, however, subjects with a sleeping problem were not included. Hence, the correlation between the assumed and actual sleeping hours was high (r=0.99) and the difference between the 2 objective sleeping hour measurements was small. Sleep efficiency, one of the indices representing sleep quality, was high, 90.5 % on average. If subjects with a sleeping problem had been included, the correlation and difference between reported and assumed or actual sleeping hours would have been weaker and larger, respectively. This is because the measurement of actual sleeping hours was calculated from assumed sleeping hours and the sum of epochs being scored as awake during sleep, which indicates that actual sleeping hours may largely reflect sleep quality. Furthermore, in considering the biological mechanisms responsible for the development of psychiatric and somatic problems caused by short sleeping hours, recent studies have mentioned that sleep quality as well as quantity play a crucial role in the development of such diseases through changes in hormonal secretion and autonomic nerve activity^11^^,^^12^^)^. For instance, nocturnal growth hormone secretion starts immediately after the onset of sleep and is secreted markedly in the first half of the night, during which slow wave sleep predominates in EEG^11^^,^^21^^)^. In addition to a decrease in sleep quantity such as caused by sleep deprivation, a decrease in sleep quality such as fragmented sleep, as seen in patients with obstructive sleep apnea syndrome, results in a remarkable decrease or absence of GH secretion^11^^,^^21^^,^^22^^)^. Sympathetic nerve activity, evening cortisol secretion, and glucose tolerance are also dependent on sleep quality and quantity^12^^)^. Thus, the measurement of actual sleeping hours may be a more important index in subjects with sleeping problems than assumed sleeping hours. Therefore, reported sleeping hours should be cautiously used in a survey that includes subjects with sleeping problems.

As a limitation of the present study, this study included a relatively small number of subjects and may have tended to include subjects of parents who were interested in the sleeping habits of their children. The validity of reported sleeping hours in a general population might be lower than that found in the present study. Furthermore, these parents might have paid particular attention to the sleeping habits of their children during the study periods. These factors may affect the accuracy of the sleeping hours of their children. Further study is necessary, therefore, to clarify a more accurate assessment of the validity of sleeping habits of young children as reported by their parents.

In conclusion, the sleeping hours of young children as reported by their parents was strongly associated with objective sleeping hours. Furthermore, although parents tended to overestimate the sleeping hours of their children, the difference between the reported and objective sleeping hours was not so large. These results indicate that the sleeping hours of healthy young children as reported by parents could be used in a survey that required data of relative differences in sleeping hours amongst a given population.
